# From Cervical High-Grade Squamous Intraepithelial Lesion (HSIL) to Term Delivery: A Case of Pregnancy After Trachelectomy With Cerclage

**DOI:** 10.7759/cureus.101314

**Published:** 2026-01-11

**Authors:** Maria Borges Oliveira, Margarida Figueiredo, Ana Marta Pinto, Marisa Moreira, Helena Nascimento

**Affiliations:** 1 Obstetrics and Gynaecology, Unidade Local de Saúde da Região de Aveiro, Aveiro, PRT

**Keywords:** cervical cerclage, fertility-sparing surgery, high-grade squamous intraepithelial lesion (hsil), reproductive age women, trachelectomy, uterine cervical cancer

## Abstract

Cervical cancer remains one of the most common malignancies affecting women, with a significant proportion of cases diagnosed during reproductive age. Fertility-sparing approaches, such as radical trachelectomy, have emerged as viable alternatives to radical hysterectomy in selected early-stage cases, allowing preservation of reproductive potential without compromising oncological outcomes. The placement of permanent cerclage has been associated with improved obstetric results. We present a case of a nulliparous woman in her 30s initially managed for persistent high-grade squamous intraepithelial lesion (HSIL), corresponding histologically to cervical intraepithelial neoplasia grade 2/grade 3 (CIN2/CIN3), despite multiple conizations with clear margins. Following oncological referral, she was diagnosed with FIGO ( International Federation of Gynecology and Obstetrics) stage 1B1 cervical cancer and underwent radical trachelectomy with colpectomy, pelvic lymphadenectomy, and placement of a permanent cerclage. Two years later, she conceived spontaneously. Apart from an episode of threatened preterm labor at 28 weeks requiring surveillance and fetal lung maturation, the pregnancy progressed without other major complications. An elective caesarean section at 37 weeks resulted in the delivery of a healthy full-term neonate. This case illustrates the potential for successful term pregnancy following fertility-sparing surgery with permanent cerclage in carefully selected patients, emphasizing the importance of multidisciplinary management and close obstetric surveillance.

## Introduction

Cervical cancer remains one of the most common cancers among women, ranking as the ninth most frequent cancer in women in Europe [1,2]. Since nearly half of these cases affect women of reproductive age, providing fertility-sparing options without compromising oncological outcomes is essential [1,2].

Historically, radical hysterectomy and radiotherapy were the number one approaches in early-stage cervical cancer, both of which result in irreversible loss of fertility. Over the past three decades, fertility-sparing surgical techniques have been developed for appropriately selected patients. Radical trachelectomy has emerged as a viable alternative, preserving the potential for future pregnancies without compromising oncological outcomes [3]. Furthermore, associated with a permanent cerclage, it has been shown to improve obstetric outcomes, particularly by reducing the risk of mid-trimester pregnancy loss and increasing the chances of term delivery.

Persistent HSIL is associated with an increased risk of progression to invasive disease, particularly when recurrent despite adequate excisional treatment [4]. In women with early-stage cervical cancer who desire future fertility, radical trachelectomy has become an accepted oncologic option [3]. However, reproductive and obstetric outcomes, such as cervical insufficiency and preterm birth, remain key concerns and must be considered when counseling patients undergoing fertility-sparing treatment [5].

## Case presentation

We present a case of a nulliparous woman in her 30s followed in a cervical pathology clinic after an abnormal cervical cancer screening showing low-grade squamous intraepithelial lesion (LSIL). At that time, human papillomavirus (HPV) testing was not available.

Following cervical vaporization, persistent HSIL was identified. The patient completed HPV vaccination, and a first cervical conization was performed, revealing a cervical intraepithelial neoplasia grade 2 (CIN2) lesion with negative margins. During follow-up, persistent HSIL with HPV 16 positivity led to two additional conizations. Despite the absence of visible lesions on colposcopy, histopathological analysis of both specimens revealed CIN2/CIN3 lesions with clear margins.

Given persistent high-grade disease after three conizations, the patient was referred to a gynecologic oncology center for a second opinion. After multidisciplinary evaluation, she was diagnosed with FIGO (International Federation of Gynecology and Obstetrics) stage IB1 cervical cancer [6]. Tumor size, depth of stromal invasion, lymphovascular space invasion, and margin status were assessed, with no evidence of lymph node involvement.

**Figure 1:**
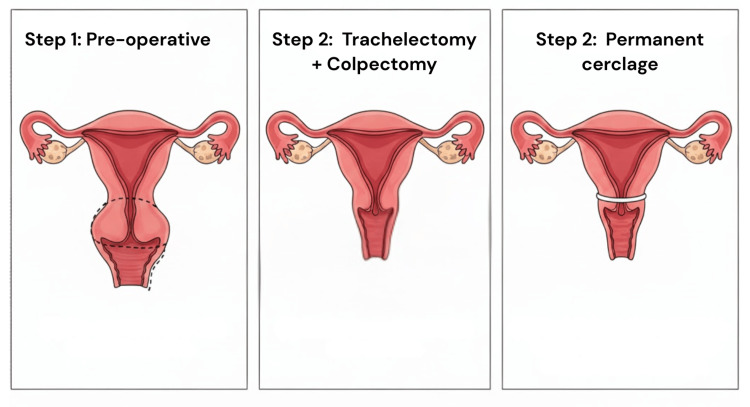
Schematic representation of radical trachelectomy with permanent cerclage, illustrating preservation of the uterine body Image Credit: Authors

The patient subsequently underwent abdominal radical trachelectomy with colpectomy, pelvic lymphadenectomy, and placement of a permanent cerclage at the level of the internal os. A schematic representation of the surgical procedure is shown in Figure 1. Histopathological examination of the surgical specimen confirmed FIGO stage IB1 disease with no residual tumor. 

Two years after surgery, at the age of 36 years, the patient conceived spontaneously. The pregnancy was monitored in a high-risk obstetric setting, with measurement of the residual cervical length every two weeks after 24 weeks of gestation and intravaginal progesterone since early pregnancy. Apart from an episode of threatened preterm labor at 28 weeks, requiring bed rest, inpatient surveillance, and fetal lung maturation, no other major complications were observed. An elective cesarean section was planned due to the history of radical trachelectomy with permanent cerclage and was performed at 37 weeks without complications. A healthy male neonate weighing 3200 g was born, with an Apgar score of 9 at one, five, and 10 minutes [7].

Currently, eight years after surgery, she maintains an up-to-date cervical screening, with a negative co-test. 

## Discussion

Beyond the anatomical ability to conceive, reproductive outcomes and pregnancy success are also increasingly recognized as critical endpoints when evaluating fertility-sparing strategies. Available evidence suggests that many women who attempt to conceive after radical trachelectomy may achieve full-term pregnancies, especially if cervical cerclage is also performed. There is an obvious increased risk of cervical insufficiency and preterm labor in this population, which is why the realization of concomitant cerclage is so relevant. The possibility of resorting to assisted reproduction procedures should also be offered, as it may play a crucial role in select cases [8,9].

## Conclusions

This case illustrates the potential for successful term pregnancy following radical trachelectomy with permanent cerclage in carefully selected patients. However, fertility-sparing surgery is associated with significant obstetric risks, including cervical insufficiency and preterm birth, and outcomes from a single case should not be generalized. Multidisciplinary follow-up and individualized counselling remain essential to optimize both oncological safety and pregnancy outcomes. 

## References

[1] Bruni L, Albero G, Serrano B (2023). ICO/IARC Information Centre on HPV and Cancer (HPV Information Centre). Europe: Human Papillomavirus and Related Diseases Report. Human Papillomavirus and Related Diseases Report: Europe.

[2] Wang ZT, Dang CW, You RL, Li S, Jiang TT, Zhu FB, Gu CL (2025). Age-period-cohort analysis of global, regional, and national cervical cancer prevalence trends in women of childbearing age (15-49 years), 1990-2021. BMC Womens Health.

[3] Martínez-Chapa A, Alonso-Reyes N, Luna-Macías M (2015). Reproductive results of radical trachelectomy (Article in Spanish). Ginecol Obstet Mex.

[4] Perkins RB, Guido RS, Castle PE (2020). 2019 ASCCP risk-based management consensus guidelines for abnormal cervical cancer screening tests and cancer precursors. J Low Genit Tract Dis.

[5] Fan H, Su X, Yang B, Zhao A (2017). Aryl hydrocarbon receptor and unexplained miscarriage. J Obstet Gynaecol Res.

[6] Bhatla N, Denny L (2018). FIGO cancer report 2018. Int J Gynaecol Obstet.

[7] AP V (1953). A proposal for a new method of evaluation of the newborn infant. Curr Res Anesth Analg.

[8] Kim CH, Abu-Rustum NR, Chi DS (2012). Reproductive outcomes of patients undergoing radical trachelectomy for early-stage cervical cancer. Gynecol Oncol.

[9] Plante M, Renaud MC, Hoskins IA, Roy M (2005). Vaginal radical trachelectomy: a valuable fertility-preserving option in the management of early-stage cervical cancer. A series of 50 pregnancies and review of the literature. Gynecol Oncol.

